# The intricate inner workings of the psychooncology department

**DOI:** 10.1192/j.eurpsy.2026.11551

**Published:** 2026-07-31

**Authors:** J. Knific, S. Kocijančič Azzaoui

**Affiliations:** Psycho-oncology department, Institute of Oncology Ljubljana, Ljubljana, Slovenia

## Abstract

**Introduction:**

The Psycho-Oncology Department at the Institute of Oncology in Ljubljana was established in 1984. It functions as an outpatient and consultative psychological–psychiatric service rather than a separate department. It provides support to oncology patients and their relatives during both outpatient treatment and hospitalization.

**Objectives:**

The paper presents the organization, developement and workings of services at a specialized psycho-oncology department.

**Methods:**

The service addresses psychological and psychiatric distress related to cancer diagnosis, treatment, survivorship, recurrence, and other associated challenges.

**Results:**

Each day begins with a team meeting and triage of in-hospital referrals from the previous 24 hours, followed by individual patient referrals and internal team referrals (between psychology and psychiatry). Each specialist has their own outpatient office for oncology patients and, when needed, conducts consultations on various wards (surgery, intensive care, radiotherapy, internal medicine, and the palliative care unit). The department also participates in multidisciplinary teams for palliative care, clinical nutrition, pain management, and comprehensive rehabilitation. There is an ongoing eight-session, CBT-based group program for cancer patients who have completed acute treatment. Occasionally, two groups are conducted simultaneously, depending on referral numbers. A recent addition is a semi-structured support group for patients in the metastatic stage of the disease. Educational activities are continuously ongoing. The department provides supervision for residents rotating through psycho-oncology as part of their training, while also contributing to formal teaching at the Faculty of Medicine and delivering specialized lectures for psychologists and other healthcare professionals. Another new initiative this year is the introduction of Balint groups for oncology residents, which the department hosts and co-moderates. Several educational brochures have been published, along with a highly productive collaboration on an educational mobile application for breast cancer patients. Whenever time permits, the team also engages in research activities and regularly participates in national and international congresses.

**Conclusions:**

The Psycho-Oncology Department in Ljubljana, currently staffed by two psychiatrists, two clinical psychologists, and a highly skilled nurse, offers a wide range of activities that continue to evolve and expand over time.

**Disclosure of Interest:**

None Declared

